# Disposable Screen Printed Electrochemical Sensors: Tools for Environmental Monitoring

**DOI:** 10.3390/s140610432

**Published:** 2014-06-13

**Authors:** Akhtar Hayat, Jean Louis Marty

**Affiliations:** 1 Images, Université De Perpignan Via Domitia, 52 Avenue Paul Alduy, Perpignan Cedex 66860, France; 2 Interdisciplinary Research Centre in Biomedical Materials (IRCBM), COMSATS Institute of Information Technology (CIIT), Lahore 54000, Pakistan; E-Mail: akhtarloona@gmail.com

**Keywords:** screen printed electrode, fabrication methods, sensors, electrochemical detection, environmental investigation

## Abstract

Screen printing technology is a widely used technique for the fabrication of electrochemical sensors. This methodology is likely to underpin the progressive drive towards miniaturized, sensitive and portable devices, and has already established its route from “lab-to-market” for a plethora of sensors. The application of these sensors for analysis of environmental samples has been the major focus of research in this field. As a consequence, this work will focus on recent important advances in the design and fabrication of disposable screen printed sensors for the electrochemical detection of environmental contaminants. Special emphasis is given on sensor fabrication methodology, operating details and performance characteristics for environmental applications.

## 1. Introduction

A major part of analytical research activity is devoted to the development of new and robust methodologies. For example, new analytical tools are required for economical and real time monitoring of environmental pollutants, and for prevention of toxic materials in the environment. Progress in the field of analytical chemistry is aimed at bringing the analytical data close to the production operations [1–3]. Such advances offer improved analytical methods with reduced environmental impact. A real time field detection system is highly desirable for continuous environmental monitoring to overcome the limitations such as sample collection and transport to a central laboratory, problems associated with commonly used methods for environmental pollutants. In this context, real time methods offer a rapid return of the chemical profile (alarm tools for sudden discharge) with minimized errors and costs as compared to the offsite laboratory-based analyses [1]. The development of portable approaches and devices with reduced sample volume is of considerable interest for both centralized and decentralized (field) analyses. This review paper highlights recent advances, primarily from the authors' laboratories, aimed at designing electrochemical systems for meeting the needs of analytical chemistry. Electrochemical devices offer unique properties to address the challenges of analytical chemistry. The advantages of electrochemical devices include possibility of miniaturization and portability, sensitivity, selectivity, a wide linear range, minimal space and power requirement and cost effective instrumentation. Devices based on the electrochemical detection are well established for many years. The past decades have seen enormous progress in electro-analytical chemistry with the development of ultra-microelectrodes, tailored interfaces, molecular devices and smart sensors. These developments have resulted in substantial popularity of electro-analyses and to their expansion into new phases and environments [4,5].

As we enter the 21 century, we do not want to rely on cumbersome electrochemical cells and bulky electrodes but rather would like to have fast, small, easy to use, portable, economical and disposable electrode systems. A vast array of electrodes for on site and *in situ* environmental monitoring has been developed during recent years [6–10]. Several representative examples, illustrating the scope, power, versatility and application of such miniaturized electrodes for environmental monitoring are described in the coming sections. This review paper as whole will focus on the trends in screen printed electrode design, screen printed electrode fabrication processes, types of screen printed electrodes and finally their environmental applications.

## 2. Towards Easy to Use, Disposable and Portable Screen Printed Electrodes

**Figure 1.**
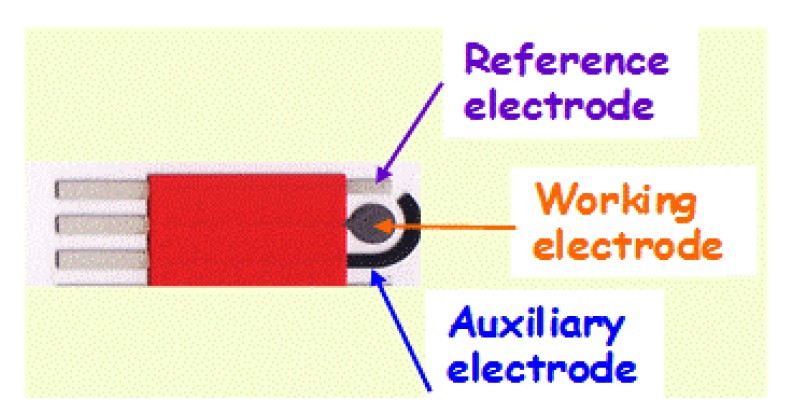
Design of a disposable and portable screen printed electrode (with reference, working and auxiliary electrodes on the same substrate) (IMAGES, Perpignan, France).

The elimination of bulky materials and instruments from the analytical protocol is a major thrust of analytical chemistry. The performance of analytical methods is directly related to the material of the working electrode. For many years, mercury was used as the most suitable electrode material due to its very attractive behavior and highly reproducible, renewable and smooth surface. These distinct properties of the mercury drop electrode led to the Nobel Prize in Chemistry awarded to Heyerovský in 1959. Both doping and hanging electrodes have been widely used in various polarographic and electrochemical techniques [1]. With the advancements in electro-analytical science, various non-mercury electrodes have also been examined. For example, bismuth and carbon electrodes started to be used in electro-analysis more than three decades ago due to their low background current, wide potential range, chemical inertness and suitability for various sensing and detection applications [11–13]. Recently, miniaturization of the solid electrodes was used to get several fundamental and practical advantages including such as a dramatic reduction in sample volume, portability and cost effectiveness. To address the needs of on-site analysis, it was necessary to move away from the commonly used cumbersome electrodes and cells. The exploitation of new fabrication techniques allows the replacement of traditional beaker type electrochemical cells and bulky electrodes with easy to use sensors. Fabrication of printed devices on bendable substrates has enabled the development of a wide range of new electrode systems. Screen printing technology is a well established technique for the fabrication of economical, portable and disposable electrode systems [14,15]. The whole electrode system, including reference, counter and working electrodes can be printed on the same substrate surface [8] (Figure 1). One prominent commercialization of screen printed electrode is the glucose biosensor used for diabetes which represents a billion dollar per year global market [16–18]. Society is in constant state of growth and development, and it is evident that demands for sensing devices related to the environment will increase with the passage of time. In order to achieve this, accurate, portable and rapid devices are highly needed. Decentralized analyses are necessary and thus traditional analytical methods cannot cope with these requirements.

Screen printed electrodes not only address the issue of cost effectiveness but also satisfy the requirement of portability, a progress towards decentralized analysis. The adaptability of screen printed electrodes is of vital importance in the area of research, the ability to modify electrodes with ease through different inks commercially available for the reference, counter and working electrode, allows for highly specific and finally calibrated electrodes to be produced for specific target analytes [8,19,20]. Many kinds of screen printed electrode modifiers exist for environmental monitoring such as noble metals, inorganic nanomaterials, proteins, enzymes and DNA sequences [21]. Screen printed electrodes combine the properties of ease of use and portability with simple, inexpensive analytical methods [22,23]. Consequently, screen printed electrodes can be easily adapted to *in situ* environmental monitoring to achieve improved performance, as has been demonstrated over the past several years.

## 3. Working Principle of a Screen Printed Electrochemical Sensor

**Figure 2.**
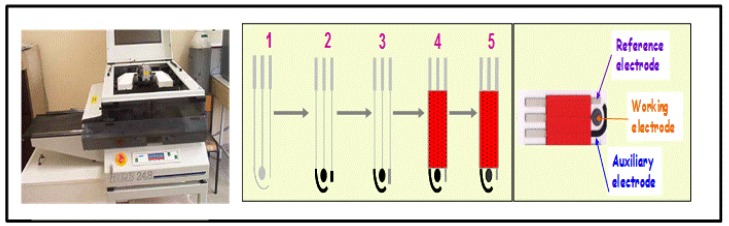
Fabrication of a three electrode system. Chemically inert substrate; screen printing of working and auxiliary electrode; screen printing of reference electrode; screen printing of protection paste; working electrode incubation with the analyte of interest (IMAGES, Perpignan, France).

Screen printed methodology offers an attractive way to design new generation electrochemical sensors. Scientists from across different fields have shown their interest in designing low cost and reliable screen printed electrochemical sensors. A detailed description of screen printed electrode fabrication methodology was already reported in a review paper by Li *et al.* [9]. Briefly, a screen printed electrode comprises a chemically inert substrate on which three electrodes, including working electrode, reference electrode and counter electrode, are printed through screen printing methodology. The working electrode is the principal electrode on which electrochemical reactions are performed, while the reference electrode and counter electrode are used to complete the electronic circuit. Figure 2 represents the stepwise fabrication of a screen printed electrode. The chemical or biological event on the screen printed electrode is converted into a detectable signal with the integration of a transducer element. Among the different transduction techniques, the electrochemical method of detection has attracted more interest for the design of low cost devices. Electrochemical methods of detection include amperometric (based on the current measurement), potentiometric (based on the voltage or potential differences) and conductometric (based on the conductivity or resistance). Among the electrochemical sensing techniques, amperometric detection is widely adopted due to its high sensitivity and applicability.

The fabrication of an electrochemical screen printed sensor usually involves three steps: fabrication of the screen printed electrode, surface design of the screen printed electrode and subsequently utilization for a sensing application. The next section will focus on the fabrication strategies employed to design efficient electrochemical sensors in term of analytical characteristics.

## 4. Dynamics of Screen Printed Electrochemical Sensors

The inks used in screen printed electrode fabrication consist of particles, polymeric binder and other additives for improved dispersion, printing and adhesion process. The exact ink formulation and composition are patented by the respective companies, and are not disclosed to the users. The variation in the ink composition such as types, size or loading of particles strongly influence the electron transfer process and change the overall performance of the designed screen printed sensor [24–27]. However, screen printed electrodes surface can be very easily modified with a variety of materials and structurally related materials to compensate these limitations. Although these screen printed sensors have found widespread applications, the fundamental understanding of the electrochemical reactivity at the screen printed electrode is still rarely studied and addressed. In this regards, Sljukie *et al.* have shown that the performance of a macro-screen printed sensor can be improved by the use of ultrasound through increase in mass transport of the analyte and removal of surface active species. [28] Choudhry *et al.*, have for the first time explored the fact that the electrode morphology can be changed dramatically by varying the concentration of the polymeric binder [29]. The same group has demonstrated that a bespoke screen printed electrode can be modified with electro-active palladium for the electrochemical oxidation of hydrazine where the unmodified electrode exhibited slow electron transfer [30]. It has also been shown that non-linear diffusion over an electrode surface may affect the individual contributions of edge and basal plane materials, affecting the relative area of the electrode surfaces [31]. Recently, Choudhry and Banks have designed a screen printed electrode substrate modified with nickel nanoparticles supported on boron diamond. These nickel-modified electrodes were shown as a potential analytical tool for the detection of alcohols [32].

The other fundamental expect of understanding screen printed electrodes reactivity is to explore the creation of surface oxygen functionalities, and the use of mediators on the electrode surface [4]. The next section will highlight some of the important advances made on screen printed electrodes creating oxygen functionalities and edge plane like sites, along with some chemical modifications.

### 4.1. Preanodized Screen Printed Carbon Electrode

The research on several carbon materials, especially carbon nanotubes and graphene, has revolutionized the scope of screen printed electrodes in electro-analysis. Compton's group observed enhanced electrocatalytic properties of CNT towards several target analytes attributed to the defect/edge plane-like sites [33,34]. Zen *et al.* demonstrated the creation of defect/edge-like sites and oxygen functionalities on the screen printed electrode surface through a simple pre-anodization process. Prasad *et al.* performed a comparative study on the role of oxygen functionalities and edge plane sites created achieved through a pre-anodization process and oxygen plasma treated screen printed electrodes [4]. Consequently, the pre-anodized electrode showed better characteristics in terms of reduction in overpotential and separation of oxidation peaks for the detection of uric acid and dopamine compared to the oxygen plasma-treated screen printed electrodes [35,36]. Similarly, the versatility of the pre-anodized screen printed electrode has been demonstrated in different media for enzymeless detection, detection of poorly electro-active analytes and direct electron transfer-related researches [37,38].

### 4.2. Mediator Integrated Screen Printed Electrode

There are many target analytes which have no significant electroactivity or near impossible to get electrochemical signals. In such cases, the use of an electrocatalyst mediator and methodologies to improve the sensor performance are very common. The appropriate selection of the mediator can improve the selectivity and lower the working potential for electrocatalysis. Redox mediators such as metal/metal complexes and pure organic polymers can be used as electrode modifiers. The viable approaches to immobilize the mediator on the screen-printed electrode include drop casting, physical attachment, and covalent binding or mixing into carbon paste. The mediator mixing approach has found more applications, and has been the mainly used technique for many decades, since the pioneering work of Adams *et al.*, Various types of mediators such as Meldola's Blue, Prussian Blue, crown ethers, cobalt phthalocyanine and nickel hexacyanoferrate have been successfully integrated into the screen printed ink to design sensors for many target analytes. Ionic liquids have been used in analytical chemistry and carbon composites due to their physiochemical properties and biocompatible nature [39,40], but they were never been used in screen printed electrodes fabrication until the notable work by Ping *et al.*, who incorporated a variety of ionic liquids into screen printed electrodes [41]. This work was further extended by Ren *et al.* in the fabrication of DNA sensors to achieve nano-level sensitivity [42]. Recently, carbon nanotubes-mediated screen printed electrodes have been used to increase the electrochemically active area of screen printed electrodes, subsequently employed in the detection of *p*-aminophenol [43]. This work has provided a base to use other carbon nanotubes such as single walled carbon nanotubes and multi-walled carbon nanotube derivatives in designing screen printed carbon electrodes, with the possibility of accessing mass produced and reproducible nanotube-modified screen printed electrodes.

### 4.3. Metal Oxide Based Screen Printed Electrodes

Despite their various advantages, mediator approaches have the drawback of instability, and the fact they are not easily mass produced reproducibly, posing the problem of optimization of the sensors. As an alternative, metal oxides, including ruthenium oxide, copper oxide, nickel oxide, manganese oxide and bismuth oxide, have been used in the modification of electrodes [8,44], and the subsequently modified electrodes were used for many sensing applications. Among all the oxides, bismuth oxide is well documented to enhance the electro-analytical performance of the sensors, with reduced toxic effects. Since the pioneering work of Wang *et al.*, bismuth-modified electrodes have been extensively explored for diverse applications. Bismuth nanopowder immobilized with Nafion, and electrochemically oxidized bismuth oxides are normally used to obtain a uniform layer of bismuth on the electrode surface. The electrochemical deposition of the target species on the bismuth layer rather than the underlying graphite electrode results in the improved analytical performance of the sensors. Bismuth domains, as preferential nucleation sites, are distributed across the electrode surface producing their own diffusion zones and minimizing the effect of surface coverage which may result in an increased electron transfer resistance thus reducing the sensor sensitivity [45,46]. An advantageous approach for screen printed electrode modification is the one in which any metal oxide can be readily incorporated onto an electrode surface, allowing a true platform technology.

## 5. Screen Printed Sensors for Environmental Monitoring

**Figure 3.**
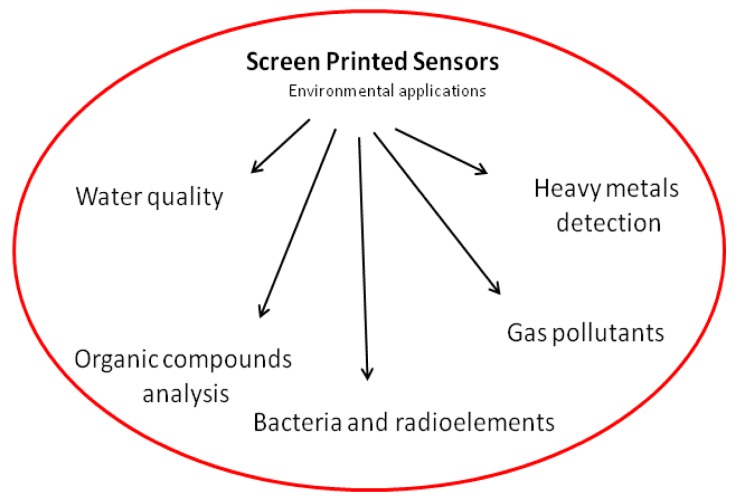
Potential environmental applications of screen printed sensors.

Screen printed electrodes have been employed as a tool to design disposable and portable electrochemical sensors for environmental monitoring, such as water quality tests, organic compound analyses, heavy metals detection and gas pollutants (Figure 3).

### 5.1. Water Quality Tests

It is of vital importance to monitor source water and the aquatic systems that can be contaminated by industrial waste, sewage treatment plants and runoff from urban and agricultural lands. Water quality monitoring is mainly based on the measurement of the physical, chemical and bacteriological characteristics of water. The physical elements of analysis include monitoring of temperature, pH and conductivity, while chemical analyses measure oxygen, alkalinity, nitrogen and phosphorus compounds. Thousands of emerging contaminants may be present in water resources, including those used for drinking water production, therefore, the development of cost-effective devices for on-line and continuous monitoring of water quality is highly desirable. In this context, the use of screen printed electrodes to monitor pH changes has attracted great attention to replace the commonly used methods for testing pH changes. Koncki *et al.*, designed a plastic, fully screen-printed, disposable pH sensor based on ruthenium dioxide by the application of a thick-film technology. The electrodes enable fast measurements, with good sensitivity in acidic and neutral solutions [47]. pH sensors with both printed reference and working electrodes on one substrate are reported in the literature [46], however this concept led to printing of the three electrode system for pH sensing with enhanced sensitivity as compared to the two electrode system [48]. Kampouris *et al.* designed a screen printed pH sensor by incorporating the pH sensitive phenanthraquinone moiety which undergoes a Nernstian potential shift with pH, and the pH insensitive dimethylferrocene one which acts as an internal reference. This generic approach offered a calibration-less and reproducible approach for portable pH measurements with the possibility of miniaturization, allowing incorporation into existing sensing devices [49]. Betelu *et al.*, investigated the applicability of CeO_2_-based screen printed electrodes for monitoring the pH of the COx pore water. However, this study was limited to the pH range between 5.5 and 13.2 [50]. Xiong *et al.* designed a calibration-less sensor based on nitrosophenyl-modified edge plane pyrolytic electrodes and screen printed electrodes to monitor pH changes [51]. Dissolved oxygen concentration is also another parameter that can be employed to test water quality. Zen *et al.*, developed an efficient photocatalytic amperometric sensor for the determination of dissolved oxygen in phosphate buffer solution using a disposable copper-plated screen-printed carbon electrode. Real sample assays for groundwater and tap water were also consistent with those measured by a commercial dissolved oxygen meter [52]. Various modified screen printed sensors have been demonstrated as potential candidates to measure the chemical oxygen demand and biochemical chemical oxygen demand for various environmental studies [53].

Nitrate is also an important analyte for environmental and human health monitoring thus its detection and quantification is very important. In this regards, some screen printed electrodes have been designed and used to detect low levels of toxic ions. Moreover, microelectrodes in combination with screen printing technology have been employed to measure the nitrate level in water samples [54,55]. For example, Lin *et al.*, fabricated poly (3,4-ethylenedioxythiophene) and PEDOT/multi-wall carbon nanotubes (PEDOT/MWCNTs)-modified screen-printed carbon electrodes (SPCEs) and studied their catalytic properties for nitrite measurement. The developed sensor was also applied to the determination of nitrite concentration in tap water samples [56]. Monchindu *et al.*, electropolymerised aniline doped with polyvinyl sulphonate on screen printed carbon electrodes. The designed electrochemical sensors exhibited good analytical characteristics for nitrate detection [57]. Lin *et al.*, investigated the oxidative electrochemistry of nitrite on a poly(3,4-ethylenedioxythiophene)/iron phthalocyanine/multi-wall carbon nanotubes-modified screen-printed carbon electrode. The developed sensor was also applied for the determination of nitrite in tap water samples [58]. Metters *et al.* reported the fabrication of screen printed graphite micro-band electrodes which were electrochemically characterized and critically explored in electro-analytical applications for the sensing of nitrite [55] .Saljukis *et al.*, fabricated manganese dioxide screen printed graphite electrodes for electro-analytical sensing purposes. The prepared sensors exhibited attractive performances as electrocatalysts for the sensing of nitrite ions with detection limits comparable or lower than those obtainable with other electrochemical sensors [59].

Similarly, the hydrophilic nature of phosphate ions makes them difficult to detect in water analysis. Ion selective electrodes, due to their ability to measure various species in turbid and colored medium, have been appeared as the prominent tools to measure phosphate ions for routine water sample analysis. Screen printed carbon paste and conventional PVC membrane electrodes have been integrated in ion selective sensors for phosphate ion analysis [60]. Some of the recently developed screen printed sensors for water quality are listed in Table 1.

### 5.2. Organic Compounds

Phenols are organic compounds broadly employed in the chemical, petrochemical, pharmaceutical, pesticide, pulp and paper, textile, metallurgic, resin and plastic, and pulp and paper industries. Phenol poisoning by skin absorption, inhalation of vapors or ingestion causes accumulation and damage to the brain, kidneys, liver, muscle, and eyes, as well as necrosis [21]. Therefore, detection of phenolic compounds and their derivatives is highly desirable to meet the corresponding environmental challenges. Even though the standardized methods are able to obtain accurate results for a wide range of phenolic compounds, conventional approaches are time-consuming and cost-intensive. Furthermore, they require large volumes of toxic organic solvents such as methylene chloride, acetone, and methanol. Consequently, there is a demand for the development of reliable, portable, sensitive, simple and cost-effective methods for the fast detection of phenolic compounds. Electrochemical sensors based on screen printed electrodes have been used as low cost, simple, sensitive and disposable tools for *in situ* monitoring of phenolic compounds. The possibility of direct electrochemical oxidation of these phenolic compounds at the screen printed electrode facilitates their detection [68], Many modification strategies and immobilization methods have been reported in the literature to design innovative electrochemical sensors for monitoring phenolic compounds [69–72].

Pesticides are released intentionally into the environment, and through various processes can contaminate the environment. Although pesticides are associated with many health hazards, there is a lack of monitoring of these contaminants. Traditional chromatographic methods—high-performance liquid chromatography, capillary electrophoresis, and mass spectrometry—are effective for the analysis of pesticides in the environment, but have certain limitations such as complexity, time-consuming sample preparation, and the requirement of expensive apparatus and trained persons to operate them. Over the past decades, acetylcholinesterase (AChE) inhibition-based biosensors have emerged as simple, rapid, and ultra-sensitive tools for pesticide analysis in environmental monitoring, food safety, and quality control. These biosensors have the potential to complement or replace the classical analytical methods by simplifying or eliminating sample preparation and making field-testing easier and faster with a significant decrease in cost per analysis [73,74]. Based on the inhibition mechanism of the pesticide, various electrochemical biosensors based on screen printed electrodes have been constructed to analyse water and soil samples for the presence of pesticides. Prussian Blue, carbon nanotubes, cobalt phthalocyanine and conductive polymers have been successfully integrated as mediators in screen printed electrochemical biosensors for pesticide detection [75–78].

Despite the use of modern, less persistent agrochemicals, herbicide residues and herbicide metabolites in water are a serious environmental problem. Even when used appropriately, water soluble herbicides can be found in surface waters, ground waters, and tap water. For this reason, the monitoring of herbicides and herbicide metabolites is important to ensure the quality of water. Electrochemical immunosensors based on screen printed carbon electrodes are used for single shot determination of herbicides, eliminating the cleaning and reuse of components [79,80]. However, the immunoassays undergo some drawbacks such as the time consuming antibody production process and the possibility of cross-reactivity. Alternatively, photosynthetic electrochemical biosensors based on screen printed electrodes have been proposed, and successfully implemented for herbicide detection [81–83]. Polycyclic aromatic hydrocarbons (PAHs) are a large group of organic compounds with two or more fused aromatic rings. They have a relatively low solubility in water, but are highly lipophilic. Aromatic compounds can interact with graphite walls and thus stack onto carbon materials through non-covalent binding. After concentrating polyaromatic hydrocarbons on screen printed electrodes, an operating potential can be applied for the individual electrochemical detection of a specific aromatic compound [84–86]. Similarly, immunosensor approaches based on screen printed electrodes have also been reported in the literature for a mixture of individual polyaromatic hydrocarbon compounds. Nevertheless, the very similar structures of polyaromatic hydrocarbon compounds make difficult the production of a specific antibody for only one polyaromatic hydrocarbons. Future work may focus on the integration of various antibodies within a single screen printed sensor to get different signals and detailed information regarding polyaromatic hydrocarbon mixtures. Sensitive and decentralized analysis of antibiotic residues in environmental samples is also high desirable. Tetracyclines are important classes of antibiotic that are detected by employing screen printed electrodes. Immunoassays in combination with nanoparticles on screen printed electrodes have shown great potential in the development of high sample throughput screening systems for antibiotics in environmental samples [87,88]. Table 2 provides examples of the some of the recently developed screen printed sensors for organic compounds (Table 2).

### 5.3. Heavy Metals

Due to the major negative impact of heavy metal ions toward human health and the environment, even at low concentrations, the development of simple, fast and not expensive detection methods for heavy metals is a major challenge for scientists. Among the different analytical methods for the analysis of heavy metal ions, the methods based on electrochemical sensors are widely applied for the detection of metals. Among toxic heavy metals, lead continues to be one of the most problematic. Despite considerable efforts to identify and eliminate Pb exposure sources, this metal still remains a significant health concern. Pb(II) is one of the heavy metals that has been detected with improved sensitivity by using modified carbon, bismuth, gold or other materials. These modifiers were integrated onto the surface of screen printed electrodes to make portable and disposable devices, improving their suitability for on-site analysis [100–103]. However, such approaches exhibit shortcomings such as necessity of acid or alkaline working media. Alternatively, mercury has the potential to perform analysis over a wide range of pH, and can be used as a possible screen printed electrode modifier for trace metal detections. As a proof of concept, mercury-modified screen printed electrodes have been employed to detect low levels of Cd(II) in environmental samples [104,105].

Mercury ions, the most stable form of inorganic mercury, are highly toxic environmental pollutants and have serious medical effects. Therefore, it is highly desirable to develop sensitive methods for the detection of Hg^2+^. Indeed, there have been numerous reports on optical Hg^2+^ detection by using Hg^2+^ sensitive fluorophores or chromophores, however, most of these fluorophores or chromophore-based Hg^2+^ sensors only work in organic media, which cannot be directly used to detect mercury contaminants in aqueous media. Bare gold or modified gold electrodes are normally used for the detection of Hg^2+^ due to their strong affinity for Hg^2+^ [106,107]. Commercial screen printed electrodes are reported for simple detection of Hg^2+^ in water samples. Nanostructured carbon black and screen printed electrodes modified with conducting polymer layers have also been designed for the trace level measurement of Hg^2+^ in water samples [108,109]. Arsenic is also a common compound found in drinking water, especially in some Asian countries. The toxicity of arsenic is greatly dependent on its oxidation state since As(III) is 50 times more toxic than arsenate due to its reactions with enzymes involved in human metabolism. Many detection methods have been developed for determination of such levels of arsenic. Among these methods, electrochemical methods provide accurate measurements of low concentrations of metal ions at ppb levels with rapid analysis times and low cost instrumentation. Screen printed electrodes modified with nanoparticles have been utilized to detect arsenic in water environments [110]. However, to avoid interferences from other metals, enzymatic biosensors based on screen printed electrodes for the measurement of arsenic in water samples have also been reported in the literature [111]. Designing screen printed sensors for simultaneous detection of various metals is also interesting for time and cost reasons. Screen printed electrodes modified with gold nanoparticles/gold films have also been reported for stripping voltammetric determination of mercury (II) and lead (II) [112]. Selected and recently developed screen printed sensors for heavy metals detection are listed in Table 3.

### 5.4. Gas Pollutants

The air pollution caused by exhaust gases from automobiles has become a critical issue. In some regions, fossil fuel combustion is a problem as well. The principal gases that cause air pollution from automobiles are nitrogen oxide and carbon monoxide. Conventional and traditional methods to detect the levels of toxic gases include color reactions, chemiluminescence and IR absorption approaches. In comparison to these described methods, electrochemical gas sensor based on screen printed electrodes can provide low cost, easy to use and portable devices for environmental analysis.

Carbon monoxide is a colorless, odorless, tasteless and poisonous gas mainly produced by the combustion of fossil fuels. Bare gold, nanoparticles particles-modified carbon and SnO_2_-modified carbon electrodes based on screen printing technology have been employed to detect the levels of this gas in environmental samples [130,131]. These devices have the potential to be used for *in situ* measurement and for continuous monitoring. Nitrogen oxide is a prominent air pollutant produced during high temperature combustion processes. The symptoms of nitrogen oxide poising appear several hours after its inhalation and require sensitive methods to detect it at low levels. Tin-doped and indium oxide thin films on screen printed electrodes have been used for the detection of nitrogen oxide in the air samples [132,133]. Volatile organic compounds including formaldehyde, acetone and methanol pose harmful effects to human health and contaminate the environment. Screen printed nanocomposite films integrated with multi-walled carbon nanotubes and silicon binders have been used to measure organic gases [134,135].

### 5.5. Other Environmental Pollutants

The presence of bacteria may also pose some enteric disease problems and indirectly results in economic losses. Enzyme-labeled and impedimetric immunoassays based on screen printed electrodes have been developed for bacterial detection in river and tap water samples without pre-concentration steps [136]. Screen printed micro-system have been designed for pathogen detection that serve as both functional and structural components, to improve the simplicity of the fabrication steps [137]. Radio-elements are also considered as radiological and chemical toxic compounds, with their presence in the aquatic system needs to be monitored. Screen printing technology has efficiently contributed in the detection of radioelements, and screen printed sensors to monitor uranium have been reported in the literature [138,139].

## 6. Conclusions and Future Prospects

As discussed in this review paper, there have been many exciting developments in the use of screen printing to design new types of electrochemical sensors. The combination of modern electrochemical systems with screen printing technology along with breakthroughs in micro-electronoics and miniaturization permits the introduction of powerful and potential analytical tools for effective monitoring of environmental pollutants. Such real time on-site monitoring methodologies have successfully addressed the time constraints associated with classical laboratory analysis. With the passage of time, electrochemical devices are becoming more and more sophisticated and versatile while dramatically shrinking in size and weight. Screen printed methodologies offer the advantage of production of simple, economical, disposable, portable and mass produced devices suitable for on-site analysis of environmental pollutants. Disposable screen printed electrodes have extensively improved the sensitivity and selectivity of the analytical approaches, especially in the detection of certain environmental analytes that were difficult and challenging to measure with conventional and traditional techniques. The field of screen printed electrodes, however, continues to grow and find new application domains. It is expected that future work shall focus on the integration of nanomaterials in the screen printed electrodes to improve the electron transfer rates, thus enhancing the analytical performance of the sensors. Furthermore, microchip formats may find application to improve the miniaturization process to decrease the analysis time, sample volumes and reagent consumption and enhance portability and for on-site analysis.

## Figures and Tables

**Table 1.** Some of the recently developed screen printed sensors for water quality tests.

Analyte	Modifier	Detection Method	Ref.
Liquids	Iridium and ruthenium oxide	pH sensor	[61]
Liquids	Phenanthraquinone moiety	pH sensor	[49]
Hydroxide ions	Nickel oxide bulk	pH sensor	[48]
Dissolved oxygen	CdS modified	Cathodic electrochemiluminescenc	[53]
Nitrite	Poly(dimethylsiloxane)	Amperometric detection	[54]
Nitrite	Shallow recessed unmodified	Amperometric detection	[25]
Phosphate	Bisthiourea ionophores	Amperometric detection	[60]
Nitrite	Carbon Black	Multi-electrochemical methods	[62]
Phosphate	Electrocatalyst cobalt phthalocyanine	Amperometric	[63]
Phosphate	Cobalt phthalocyanine	Amperometric	[64]
Nitrate	Modified screen printed electrodes	Electrochemical detection	[65]
Nitrate	polymer (poly(vinyl alcohol)) modified	Amperometric	[66]
Nitrate	commercial screen-printed electrochemical cell	Amperometric	[67]

**Table 2.** Examples of the some of the recently developed screen printed sensors for organic compounds detection in environmental samples.

Analyte	Modifier	Detection Method	Ref.
Organophosphate	Poly(3,4-ethylenedioxythiophene) (PEDOT)	Amprometric	[76]
Organophosphate pesticides	Cobalt phthalocyanine	Chronoamperometry	[89]
Organophosphorus	Cysteamine self-assembled monolayer	Amprometric	[90]
Organophosphorus and Carbamate Pesticides	Unmodified	Amperometry, flow system	[91]
Aminophenol isomers	Untreated SPCE	Voltammetric	[21]
Organophosphorus Pesticide	Single-walled carbon nanotubes— Co phtalocyanine	Amperometry	[79]
Organophosphorus Pesticide Dichlofenthion	Nanometer-Sized Titania	Photoelectrochemical	[81]
Herbicide isoproturon	Unmodified	Amperometric	[92]
Herbicide	Magnetic nanoparticles	Amperometric	[83]
Picric acid and atrazine	Self-assembled monolayer	Photo-electrochemical	[93]
Chlorsulfuron	Gold (Au) metal ions	Stripping voltammetry	[80]
Phenol and catechol	Bismuth nanoparticles	Amperometric measurements	[94]
Phenol and pesticide	Iridium oxide nanoparticles	Electrochemical measurement	[95]
Phenol	Carbon Black Paste	Amperometric	[96]
Phenolic compounds	Nano-HA-chitosan nanocomposite-modified gold electrode	Amperometric	[97]
Phenolic compounds	Screen-printed PEDOT:PSS electrodes	Amperometric	[98]
Carbamate Insecticide	Prussian Blue-Multi-Walled Carbon Nanotubes	Amperometric	[99]

**Table 3.** Selected and recently developed sreen printed sensors for heavy metal detections.

Analyte	Modifier	Detection Method	Ref.
Pb^2+^ and Cd^2+^	screen-printed antimony and tin	anodic stripping detection	[113]
Cu^2+^	Macrocyclic Polyamine Modified Screen-Printed Electrodes	Square wave anodic stripping voltammetry	[114]
Cd^2+^, Cu^2+^	Diazonium modified electtrodes	Amperometric detection	[115]
Pb^2+^ and Cd^2+^	Bismuth-coated	Stripping voltammetry	[116]
Pb^2+^	Reduced graphene oxide	Square wave anodic stripping voltammetry	[117]
Zn^2+^, Cd^2+^ and Pb^2+^	Multiwalled carbon nanotubes	Differential pulse stripping voltammetry	[118]
Hg^2+^ and Pb^2+^	Polypyrrole/carbonaceous nanospheres	Square wave anodic stripping voltammetry	[119]
Pb^2+^ and Cd^2+^	Bismuth–carbon nanocomposites	Differential electrochemical methods	[120]
Pb^2+^	Bismuth-antimony film	Stripping voltammetric	[121]
Pb^2+^	4-carboxyphenyl-grafted	Anodic Square Wave Voltammetry	[122]
As(III)	Gold electrode	Sequential injection/anodic stripping voltammetry	[123]
As(III)	Nanoparticles	Linear sweep voltammetric	[124]
As(III)	Modified screen printed electrodes	Amperometric	[111]
Cd^2+^, Pb^2+^, Cu^2+^ and Hg^2+^ ions	Heated graphitenanoparticle	Electrochemical stripping	[125]
Hg^2+^	Gold nanoparticles-modified	Square wave anodic stripping voltammetry	[126]
Pb^2+^, Cu^2+^ and Cd^2+^	Mercury nano-droplets	Square wave anodic stripping voltammetry	[127]
Pb^2+^	Paper disk impregnated	One-step electrochemical detection	[128]
Cd^2+^	Nafion. Cd	Square Wave Anodic Stripping Voltammetry	[129]
